# Bulk isolation of basidiospores from wild mushrooms by electrostatic attraction with low risk of microbial contaminations

**DOI:** 10.1186/s13568-017-0326-0

**Published:** 2017-01-25

**Authors:** Kiran Lakkireddy, Ursula Kües

**Affiliations:** 1 Department of Molecular Wood Biotechnology and Technical Mycology, Büsgen-Institute, Georg-August-University Göttingen, 37077 Göttingen, Germany; 2 Center for Molecular Biosciences (GZMB), Georg-August-University Göttingen, Göttingen, 37077 Göttingen, Germany

**Keywords:** *Agaricomycetes*, Fruiting bodies, Basidiospores, Isolation, Buller’s drop, Electrostatic attraction

## Abstract

The basidiospores of most *Agaricomycetes* are ballistospores. They are propelled off from their basidia at maturity when Buller’s drop develops at high humidity at the hilar spore appendix and fuses with a liquid film formed on the adaxial side of the spore. Spores are catapulted into the free air space between hymenia and fall then out of the mushroom’s cap by gravity. Here we show for 66 different species that ballistospores from mushrooms can be attracted against gravity to electrostatic charged plastic surfaces. Charges on basidiospores can influence this effect. We used this feature to selectively collect basidiospores in sterile plastic Petri-dish lids from mushrooms which were positioned upside-down onto wet paper tissues for spore release into the air. Bulks of 10^4^ to >10^7^ spores were obtained overnight in the plastic lids above the reversed fruiting bodies, between 10^4^ and 10^6^ spores already after 2–4 h incubation. In plating tests on agar medium, we rarely observed in the harvested spore solutions contaminations by other fungi (mostly none to up to in 10% of samples in different test series) and infrequently by bacteria (in between 0 and 22% of samples of test series) which could mostly be suppressed by bactericides. We thus show that it is possible to obtain clean basidiospore samples from wild mushrooms. The technique of spore collection through electrostatic attraction in plastic lids is applicable to fresh lamellate and poroid fruiting bodies from the wild, to short-lived deliquescent mushrooms, to older and dehydrating fleshy fruiting bodies, even to animal-infested mushrooms and also to dry specimens of long-lasting tough species such as *Schizophyllum commune*.

## Introduction

Fruiting bodies of *Agaricomycetes* may serve in food supply (Kües and Liu 2000) and for medicinal purposes (Wasser 2011), why isolation of mycelial cultures for mushroom cultivation is of high interest. Also, mycelia and produced enzymes might be applied in diverse fields of biotechnology (e.g. Pointing 2001; Hofrichter et al. 2010; Kües 2015a; Eibes et al. 2015; Masran et al. 2016) and further use is made of e.g. biologically active fungal polysaccharides (Cohen et al. 2002; Schmidt et al. 2011; Wasser 2011), non-enzymatic proteins (Wösten and Scholtmeijer 2015) and a broad range of secondary metabolites (Xu et al. 2010; Degenkolb and Vilcinskas 2016; Schmidt-Dannert 2016). Last but not least, Agaricomycetes have also their distinct position in fungal research, for example in studies on mating-type control of sexual reproduction (Kües 2015b), development of fruiting bodies as most complex multicellular fungal structures (Ohm et al. 2010; Stajich et al. 2010; Kües and Navarro-Gonzaléz 2015), and specific ecological functions such as by decay of lignocellulose (Floudas et al. 2012, 2015) and in mycorrhizal symbiosis (Martin et al. 2008; Kohler et al. 2015).

The typical life cycle of *Agaricomycetes* is heterothallic. It starts with meiotic basidiospores with one (1n) or two identical haploid nuclei (2 × 1n) which germinate into primary mycelia. Such a primary mycelium is called homokaryon because of the genetically identical nuclei in its cells. The more specific term monokaryon is used when primary mycelia harbour only a single haploid nucleus in their cells (1n). Primary mycelia are sterile but may fuse and, if of different mating type, will then form a fertile dikaryon. The dikaryon has binucleate cells and contains one haploid nucleus from each mating partner (1n + 1n). Mushrooms are produced on such secondary mycelia, with hymenia as specialized mycelial tissue covering their gills, ridges or pores. Dikaryotic hyphae of the hymenial layer give rise to the initially binucleate basidia with still two distinct haploid nuclei (1n + 1n). Karyogamy (2n) and meiosis (1n + 1n + 1n + 1n) occur in the basidia. Eventually, the resulting four haploid nuclei migrate individually into the four basidiospores (1n) which bud off from sterigmata at the apex of a basidium. A postmeiotic mitosis can lead to presence of two identical haploid nuclei (2 × 1n) in a spore (Kües 2000; Kües and Navarro-Gonzaléz 2015). Basidiospores of heterothallic species have different mating type specificities required for control of dikaryon, fruiting body and basidiospore development (Kües et al. 1998, 2002). However, there are also self-fertile homothallic species which form mushrooms without mating to another strain and produce basidiospores which are genetically identical to the parental strain. Pseudohomothallic species in contrast take up haploid nuclei of different mating type into their only two basidiospores (1n + 1n) per basidium, based on a nuclear mating type recognition reaction within the basidium. These heterokaryotic spores germinate then directly into fertile mycelia able to produce fruiting bodies without any further mating (Kües 2015b).

Isolation of pure mycelia from mushrooms collected from the wild may target at obtaining homokaryons or dikaryons. Basidiospores and vegetative fruiting body tissues may serve as sources for mycelium isolation (Ainsworth 1995). An obvious problem in the isolation of mycelia from the non-sterile wild mushrooms is the danger of contamination by bacteria and particularly by other fungi born from the air, soil and other surroundings. Attraction of a multitude of small animals (beetles, flies and other insects and their larvae, mites, nematodes, etc.) to the mushrooms bring in further sources of microbial contamination and very much enhances the problem. Ainsworth (1995) in his technical bulletin on methods for the isolation of basidiomycetes therefore advices to collect wild mushrooms as young as possible.

Obtaining a fertile mycelium from vegetative mushroom tissues can be difficult, depending on the size and consistency of a collected fruiting body. The risk of contamination can be reduced by semi-sterile surgery of inner parts from stipes or pilei of more compact larger mushrooms while for older, bug-ridden and small fragile mushrooms this is often impossible. Removed mushroom tissues are laid onto sterile agar medium but mycelial outgrowth can be hindered by other faster growing organisms, both bacteria and other fungi (Schuytema et al. 1966; Snelling et al. 1996; Lodge et al. 2004). In our hands, we failed in an estimated 20% of cases in isolation of mycelia from fruiting body tissues, mostly due to overgrowth by other competing microbes, especially by filamentous *Ascomycetes* or sometimes also by *Mucoromycotina* (unpublished data). Suitable antibiotics for suppression of bacteria and possibly benomyl for suppression of more sensitive *Ascomycetes* might be used. Outer tissue sterilisation prior to surgery by NaOH, ethanol or H_2_O_2_ might also reduce outgrowth of unwanted organisms. However, if isolation from mushroom tissues still fails, there might still be the possibility for heterothallic species to generate a new dikaryotic mycelium from poly-spore germination on agar plates or by mating of isolated germinated basidiospores (Schuytema et al. 1966; Snelling et al. 1996; Lodge et al. 2004). Isolation and germination of basidiospores from pseudohomothallic and homothallic species on the other hand will be sufficient to directly obtain desired fertile mycelia (Kües and Liu 2000; Kües 2015b).

Basidiospores are often collected from mushrooms as spore prints. The mushroom cap might be laid directly onto filter paper, aluminium foil or (water) agar. Alternatively, mushrooms might be fixed into the lids of Petri-dishes by agar, petroleum jelly (Vaseline) or other suitable gluing agents, or positioned by toothpicks or glass rods above the respective surfaces so that the mature spores drop down from the mushrooms onto these. Repeated spore prints might be taken from a mushroom with the hope that the number of contaminants is lower in later prints. Of advantage for many species is that basidiospores can be stored for some time. When they are plated for germination, antibiotics are then used in growth medium in order to reduce outgrowth of unwanted microorganisms as much as possible (Snelling et al. 1996; Choi et al. 1999; Lodge et al. 2004; Kropp 2005).

Basidiospores are ballistospores which are catapulted at maturity from their sterigmata at the basidia. The motion forces result from the hygroscopic Buller’s drop which grows within seconds by condensation at the hydrophobic hilar spore appendix and its rapid fusion with an also hygroscopic liquid film which arises in a dent on the adaxial side of the spore (McLaughlin et al. 1985; Webster and Davey 1985; Webster et al. 1989; Ingold 1992; Money 1998; Pringle et al. 2005). The spores are propelled into the free air space between lamellate hymenia or of a pore, fall out of the caps by gravity (Ingold 1957, 1992; Pringle et al. 2005; Money and Fischer 2009; Noblin et al. 2009; Fischer et al. 2010a), and might then be transported by air streams further to new substrates (Galante et al. 2011; Horton et al. 2013; Halbwachs and Bässler 2015; Dressaire et al. 2015, 2016).

Mushrooms with ballistospores are naturally opened to the ground which helps that the spores are falling down by gravity out of the cap (Ingold 1957, 1992). When culturing *Schizophyllum commune* dikaryons on agar medium in plastic Petri-dishes under fruiting body-inducing conditions in the lab (Ohm et al. 2010), we however repeatedly observed that masses of basidiospores accumulated against gravity in the plastic lids of Petri-dishes incubated in upright position (unpublished observations). This observation motivated experiments with wild mushrooms collected in nature. Here we demonstrate that spores can be transported against gravity out of mushrooms when these lie upside-down facing up their lamellae, ridges or pores and when electrostatic forces act on the spores. We use this observation and present a new technique of bulk basidiospore isolation from mushrooms with reduced risk of contamination by unwanted microbes. Basidiospores are attracted and attach to electrostatically charged plastic lids of sterile Petri-dishes positioned in short distance above the reversed mushrooms. Subsequently, the spores can be harvested from the lids in sterile solution for further use.

## Materials and methods

### Mushroom collection and identification

Wild mushrooms were collected as found on the North Campus of the University of Göttingen and neighbouring areas of the village of Göttingen–Weende from 09.2011 to 09.2014. Mushrooms were photographed prior to harvest using a Cannon IXUS 115 H5 digital camera (12.1 megapixels; Canon, Krefeld, Germany). Harvested mushrooms were transported into the lab and photographed again, using a ruler as size marker to allow cap size estimations. Ecological parameters of mushrooms’ growth (biotope, substrate, host trees) and morphological characters of the mushrooms (of stipes, caps, veils, lamellate hymenia or pores and, crucially in the identification process, of spores) were recorded for species determination. Basidiospores (sizes, colour and shapes) were observed under an Axioplan 2 imaging microscope (Carl Zeiss, Göttingen, Germany), photographed by a computer-linked Soft Imaging ColorView II Mega Pixel digital camera and analysed in size with the AnalySIS^®^ software program (Soft Imaging System, Münster, Germany). Averages of spore sizes of collected specimens were determined from usually 5–20 spores. The field guides of Breitenbach and Kränzlin (1986, 1991, 1995), Bresinsky and Besl (1990), Flück (1995), Dähncke (2001) and Gerhardt (2010) and the *Coprinus* pages by Uljé (http://www.grzyby.pl/coprinus-site-Kees-Uljee/species/Coprinus.htm) were used in species identifications and the MycoBank database (http://www.mycobank.org/) was considered for current species names and higher classification.

*Coprinopsis cinerea* mushrooms were all of strain AmutBmut (FGSC25122) and produced in the lab on artificial YMG/T medium (4 g yeast extract, 10 g malt extract, 100 mg tryptophan, 10 g agar) under standard fruiting conditions (Granado et al. 1997). A coincidental mushroom of *Leucocoprinus birnbaumii* was collected from a flowerpot in a student office of the institute.

### Basidiospore harvests

Any dirt and noticed animals were removed from collected mushrooms. Caps were carefully separated from stipes with a sterile razor blade and forceps. Depending on their diameter (abbreviated by *Ø* throughout this work) or on the cap height of mushrooms which never fully open their umbrellas (i.e. *Coprinopsis picacea*, *Coprinus comatus*), caps were kept intact or sliced into 2, 4 or more equally sized portions. In cases of mushrooms with thick fleshy pilei (*Amanita strobiliformis*, *Coprinopsis atramentaria*, all *Boletales* but *Hygrophoropsis aurantiaca*) the upper gill-less or tube-free pileus parts of caps were sliced off in order to generate sufficient free air space above mushrooms during the experiments in the Petri-dishes. Caps up to ca 4–5 cm in diameter or parts of caps in case of larger mushrooms (*Ø* > 4–5 cm) were laid upside-down onto sterile wet paper tissue in individual sterile plastic Petri-dishes (polystyrene, 9 cm *Ø*, with cams, REF 82.1473; Sarstedt, Nümbrecht, Germany), sterile standard glass Petri-dishes (9 cm *Ø*) or higher sterile glass dishes (9 cm in *Ø*, 3.2 cm in height; used for *Armillaria solidipes*, *Pholiota squarrosa* and *H. aurantiaca* mushrooms) covered by a lid of a plastic Petri-dish. Care was taken to ensure that there was at least 0.5 mm free airspace in Petri-dishes above the mushrooms, while the airspace between mushrooms and plastic lids on higher glass dishes were between 1 and 2 cm. Dishes were stored for a few hours to overnight (up to 18–20 h) on a bench at room temperature (RT) for spore ejection. Patterns of spores adhering to the plastic lids were photographed using a Stemi 2000-C binocular (Carl Zeiss, Göttingen, Germany) connected to the Soft Imaging ColorView II Mega Pixel digital camera. Spores attached to the lids were washed off with 200 µl sterile water or with 200 µl of sterile 0.1% Tween 80 and counted using a hematocytometer.

Directional effects of mushrooms on spore release were observed in experiments of two distinct set-ups. Fruiting bodies of a same size and age or defined parts of fruiting bodies of a species were incubated in parallel in Petri-dishes on wet tissue paper in either natural direction or in upside-down position (Experimental Set-up 1). In other experiments in order to avoid a direct contact with the wet paper tissue, mushroom samples were attached to the bases of plastic Petri-dishes by sticking them with their cap surface into a layer of sterile hand-hot water agar (1%) so that the cap surfaces touched the bottoms of the respective Petri-dishes. Petri-dishes were then incubated either in up-right position or upside-down (Experimental Set-up 2). Spores were harvested after 18 h incubation at RT either from the wet paper tissues (Set-up 1) or from the surface of the plastic lids of Petri-dishes (Set-up 2).

Two different strategies were also followed up to observe spore release over the time. First, equally sized and aged mushrooms or parts of mushrooms of a species were in parallel incubated upside-down on wet tissues in Petri-dishes at RT. At defined time points, spores were harvested from selected individual samples and counted. Data from the different individual samples for the different time points were compared (Experimental Set-up 1). In a second approach, spores were consecutively harvested in different lots per distinct mushroom sample at distinct time points of incubation. Counted spore numbers per harvest points were added together in order to obtain total spore numbers for different lengths of incubation of a given mushroom (Experimental Set-up 2).

The standard experimental set up (caps or pieces of caps laid upside down onto wet paper with plastic lids above) was changed in some experiments by using plastic Petri-dishes without tissue paper, by using plastic Petri-dishes with a layer of Vaseline smeared onto the inner lid surfaces, by using thin glass Petri-dishes with and without a layer of Vaseline smeared onto the inner lid surfaces, and by using thin transparent plastic rings from 1 to 10 cm in height and 8.95 cm in diameter as spacers between Petri-dish bases and lids in order to adjust the relative distances between reversed mushrooms positioned on wet paper tissue in the dishes and the plastic lids above.

Evaporating dishes (9 cm *Ø* 4.6 cm in height) with plastic lids above were used in experiments with gasteroid mushrooms.

### Germination tests

Spore suspensions (50 µl) as harvested were plated onto 2% MEA agar (20 g malt extract, 10 g agar) or LB agar (5 g yeast extract, 10 g tryptone, 5 g NaCl, 1 ml 1 N NaOH, 10 g agar) and incubated at 25 °C for up to 15 days. Mixtures of antibiotics (AB) were added to media as needed (end-concentrations: ampicillin 100 µg/ml, kanamycin 50 µg/ml, streptomycin 100 µg/ml, tetracycline 20 µg/ml, chloramphenicol 20 µg/ml). Plates were checked on daily basis for growth and nature of microbes. Where possible, colonies grown on a plate were counted.

## Results

### Collection of basidiospores against gravity in plastic lids of Petri-dishes

In first experiments, accumulations of spores in lids were observed when complete or bisected mushrooms of different species were overnight incubated upside-down in plastic Petri-dishes. Spores from complete or bisected mushrooms in natural orientation collected contrariwise in the bottom of plastic Petri-dishes (Table 1). In tendency after 18 h incubation at RT, spore prints of mushrooms of *Coprinellus domesticus*, *C. atramentaria*, *Lacrymaria* spec., *Paneolus papilionaceus*, *Pluteus* spec. and *S. commune* (fresh and dry specimens) incubated in natural orientation contained about tenfold more spores than harvests from spores in lids obtained after incubation of reversed mushrooms (Table 1). Thus, a considerable part of all spores in these species reacted against gravity and moved upward in the dishes. Only *Coprinellus micaceus* differed from the other species in that spore yields in both directions were comparable both after 18 h incubation and after shorter 8 h incubation tested also for this fungus (Table 1).

**Table 1:** Spore harvests in lids of plastic Petri-dishes after incubation of mushrooms in upside-down or upside-top position relative to the lids

Species	No of mushrooms per situation	Part used	No of samples	Incubation time (h)	Spore harvests	
					Mushroom upside-down	Mushroom upside-top
Experimental set-up 1: mushroom caps laid onto wet paper						
*Coprinellus domesticus*	3	1	3	18	4.8 ± 0.3 × 10^6^	6.7 ± 0.3 × 10^7^
*Panaeolus cinctulus*	2	1	2	18	4.5 ± 0.9 × 10^6^	4.0 ± 0.8 × 10^7^
*Schizophyllum commune*	3	1	3	18	7.3 ± 1.0 × 10^6^	8.3 ± 0.7 × 10^7^
*Tubaria hiemalis*	5	1	5	18	3.5 ± 1.2 × 10^4^	8.1 ± 0.9 × 10^4^
Experimental set-up 2: mushroom caps stuck into water agar						
*Coprinellus domesticus*	1	1	1	18	2.4 × 10^6^	2.1 × 10^7^
*Coprinellus micaceus* ^a^	2	1/2	3	8	1.1 ± 0.1 × 10^5^	0.9 ± 0.2 × 10^5^
				18	1.2 ± 0.1 × 10^5^	4.0 ± 0.2 × 10^5^
*Coprinopsis atramentaria*	2	1/2	3	18	2.1 ± 0.7 × 10^6^	2.2 ± 1.0 × 10^7^
*Lacrymaria* spec.	2	1/2	3	18	2.7 ± 1.2 × 10^5^	2.8 ± 1.1 × 10^6^
*Paneolus papilionaceus*	1	1	1	18	4.1 × 10^5^	3.5 × 10^6^
*Pluteus* spec.	1	1/2	1	18	6.7 × 10^4^	2.7 × 10^5^
*Schizophyllum commune* ^b^	3	1	3	18	4.7 ± 1.8 × 10^6^	6.1 ± 2.1 × 10^7^
					5.6 ± 0.8 × 10^4^	2.6 ± 0.2 × 10^5^

^a^In one series of experiments, the incubation time was stopped at 8 h to avoid faults through onset of cap autolysis. In all other instances where inkcaps were used, there was no obvious cap autolysis

^b^Spore data in the upper line are from freshly collected mushrooms, data in the lower line are from revived dry fruiting bodies after 15 days open storage incubation at RT

### Effects of humidity

We found that the amounts of spores present in lids after upside-down incubation of complete mushroom caps were influenced by humidity. There were between 2.4× and 62.1× less spores when mushrooms of the species *Coprinellus disseminatus* (50× less; 5 tested), *C. domesticus* (28.5× less; 3 tested), *Psathyrella conopilus* (40.3× less; 3 tested), *Pluteus* spec. (8.5× less; 1 tested), *Tubaria hiemalis* (2.4× less; 5 tested which were winter mushrooms soaked from snow cover) and *S. commune* (7× less; 3 tested) or halves of *C. atramentaria* (62.1× less; 1 tested) and eighths of *C. comatus* mushrooms (17.6× less; 3 tested) were incubated upside-down overnight (18 h) in dry plastic Petri-dishes as when similar sized and aged fruiting bodies or mushroom parts of a species (tested in parallel in same numbers) were laid upside-down onto wet tissues placed at the bottoms of the plastic Petri-dishes (absolute data from incubations on wet tissues were included in Table 2, absolute data from dry Petri-dishes incubations not shown). Because high humidity is required for basidiospore discharge (Webster et al. 1984b, 1989; Webster and Davey 1985; Money 1998; Noblin et al. 2009) and evaporation by the mushrooms from own tissues can provide required humidity only for a limited time (Turner and Webster 1991; Husher et al. 1999), the results indicate that transfer of basidiospores into the lids of the plastic Petri-dishes depended on an active ballistospore discharge mechanism.

**Table 2:** Spore harvests from plastic lids as covers of Petri-dishes or 3 cm-high glass jars after 18 h or 20 h (marked by *) incubation of mushrooms in upside-down position on wet paper tissues

Species	No of mushrooms	Cap *Ø* (cm)	Part used	No of samples	Spore size (μm)^a^		Spore harvests^b^
					Length	Width	
Mushrooms with gills							
*Agaricus augustus*	1	13.9	1/4	2	7.4 ± 0.5	5.0 ± 0.4	5.4 ± 2.2 × 10^6^
Agaricus bitorquis*	1	6.1	1/4	4	6.0 ± 0.4	4.5 ± 0.3	4.0 ± 1.1 × 10^6^
*Agaricus campestris*	1	4.5	1/4	4	nd	nd	4.1 ± 1.6 × 10^7^
Agaricus subfloccosus*	1	8.7	1/2	1	5.9 ± 0.4	4.8 ± 0.5	2.1 × 10^7^
*Agaricus subperonatus*	1	15.6	1/4	4	6.4 ± 0.2	4.6 ± 0.3	6.9 ± 2.1 × 10^6^
*Agrocybe dura*	4	3–3.8	1	4	12.8 ± 0.2	7.3 ± 0.1	2.6 ± 0.6 × 10^6^
*Amanita excelsa*	1	5.2	1	1	nd	nd	3.3 × 10^6^
*Amanita strobiliformis*	1	14.8	1/4	1	11.5 ± 0.7	8.5 ± 0.3	5.1 × 10^5^
*Armillaria solidipes*	5	3.2–4.6	1	5	8.5 ± 0.7	5.2 ± 0.2	2.2 ± 1.0 × 10^6^
*Conocybe tenera*	1	3.3	1	1	12.5 ± 0.1	7.2 ± 0.7	1.7 × 10^5^
*Coprinellus disseminatus*	13	1.1–1.2	1	13	8.4 ± 0.3	6.2 ± 0.2	3.3 ± 2.6 × 10^5^
*Coprinellus domesticus*	14	3.0–4.4	1	15	7.6 ± 0.4	4.5 ± 0.3	4.1 ± 1.8 × 10^6^
*Coprinellus micaceus*	6	3.4–5.6	1/2	9	8.1 ± 0.4	4.9 ± 0.1	3.4 ± 1.8 × 10^5^
*Coprinellus subimpatiens*	1	1.8	1	1	11.5 ± 0.3	6.6 ± 0.4	2.4 × 10^5^
*Coprinellus tardus*	1	3	1	1	10.5 ± 0.9	5.0 ± 0.3	4.1 × 10^5^
*Coprinellus truncorum*	1	5	1	1	8.9 ± 0.3	5.2 ± 0.1	5.3 × 10^5^
*Coprinellus xanthothrix*	2	3.8–4.0	1/2	2	7.9 ± 0.3	4.8 ± 0.2	3.1 ± 2.0 × 10^6^
*Coprinopsis atramentaria*	9	3.8–7.6	1/2	13	8.3 ± 0.6	4.8 ± 0.3	4.9 ± 2.3 × 10^6^
*Coprinopsis cinerea*	5	4.2–5.4	1	5	10.7 ± 0.4	6.8 ± 0.5	1.9 ± 0.6 × 10^5^
*Coprinopsis picacea*	1	11.6 (5.8 height)	1/2	2	14.2 ± 0.4	9.9 ± 0.4	5.6 ± 0.1 × 10^6^
*Coprinus comatus*	9	2.6–5 (4.5–9 height)	1/8	17	12.2 ± 0.5	7.6 ± 0.4	5.3 ± 2.6 × 10^6^
*Hygrocybe conica*	1	3.8	1	1	nd	nd	3.5 × 10^6^
*Hygrophorus olivaceoalbus*	1	3	1	1	11.9 ± 0.1	8.2 ± 0.2	3.9 × 10^6^
*Hypholoma fasciculare*	7	2.5–3.2	1	7	6.1 ± 0.4	3.5 ± 0.3	3.9 ± 2.6 × 10^5^
*Inocybe erubescens*	1	3.3	1	1	10.7 ± 0.3	6.8 ± 0.7	1.8 × 10^5^
*Inocybe fraudans*	1	4	1	1	9.8 ± 0.6	6.2 ± 0.2	6.4 × 10^5^
*Kuehneromyces mutabilis*	3	4.8–5.6	1/2	6	7.3 ± 0.2	4.3 ± 0.2	4.4 ± 1.7 × 10^6^
*Lacrymaria lacrymabunda*	2	5.0–5.8	1/4	4	8.9 ± 0.5	6.2 ± 0.2	4.3 ± 1.3 × 10^4^
*Lacrymaria* spec.	7	3–3.8	1	7	10.9 ± 0.3	6.0 ± 0.4	3.8 ± 1.4 × 10^5^
*Lepista nuda*	2	4.5–6.2	1/4	4	6.8 ± 0.4	4.7 ± 0.2	4.4 ± 2.0 × 10^5^
*Leucocoprinus birnbaumii*	1	5.6	1/2	2	8.8 ± 0.6	6.7 ± 0.5	2.4 ± 0.8 × 10^6^
*Marasmius cohaerens*	1	2.9	1	1	8.8 ± 0.2	4.9 ± 0.5	4.0 × 10^5^
*Marasmius oreades*	1	2.8	1	1	9.6 ± 0.6	6.3 ± 0.4	4.8 × 10^5^
Marasmius wynneae*	4	3–3.7	1	4	7.1 ± 0.3	4.2 ± 0.1	5.3 ± 0.8 × 10^6^
*Panaeolus ater*	2	2.6–3.2	1	1	12.8 ± 0.6	7.4 ± 0.5	3.8 ± 2.2 × 10^4^
*Panaeolus cinctulus*	6	3.5–3.7	1	6	13.3 ± 0.1	7.4 ± 0.3	3.1 ± 1.3 × 10^6^
*Panaeolus olivaceus*	1	3.1	1	1	12.8 ± 0.6	7.4 ± 0.5	2.3 × 10^5^
*Panaeolus papilionaceus*	4	3.6	1	4	13.6 ± 0.3	8.3 ± 0.5	3.0 ± 1.2 × 10^5^
*Panellus serotinus*	1	5.5	1	1	5.8 ± 0.1	1.9 ± 0.2	3.1 × 10^5^
*Parasola plicatilis*	2	1.4–2.6	1	2	12.1 ± 0.9	6.6 ± 0.9	4.5 ± 2.1 × 10^4^
*Pholiota squarrosa*	2	10–13	1/4	3	6.8 ± 0.1	4.0 ± 0.2	6.7 ± 2.8 × 10^5^
*Pholiota* spec.	1	6.2	1	1	nd	nd	3.4 × 10^4^
*Pholiotina vestita*	2	3.2–3.6	1/2	2	7.4 ± 0.5	5.2 ± 0.2	3.0 ± 1.2 × 10^6^
*Pluteus* spec.	2	8	2	1	nd	nd	7.1 ± 0.4 × 10^4^
*Psathyrella atrolaminata*	4	1.8–2.2	1	4	12.5 ± 0.3	7.2 ± 0.2	5.1 ± 2.4 × 10^5^
*Psathyrella candolleana*	6	3.1–5.8	1	6	7.0 ± 0.3	4.3 ± 0.4	6.2 ± 2.0 × 10^5^
*Psathyrella conopilus*	7	4–4.8	1	7	12.7 ± 0.9	6.4 ± 0.8	3.2 ± 2.3 × 10^5^
*Psathyrella microrhiza*	5	1.6–3	1	5	11.7 ± 0.3	6.5 ± 0.1	0.8 ± 0.4 × 10^4^
*Psathyrella pseudogracilis*	7	4–4.8	1	7	13.7 ± 0.8	6.6 ± 0.4	8.8 ± 2.1 × 10^5^
*Psathyrella spadiceogrisea*	1	3.8	1	1	7.9 ± 0.3	4.9 ± 0.4	8.2 × 10^6^
*Psathyrella tephrophylla*	3	4–4.4	1	3	10.5 ± 0.4	5.8 ± 0.3	2.9 ± 0.2 × 10^5^
*Russula exalbicans*	1	6.2	1	1	6.6 ± 0.2	4.7 ± 0.1	1.4 × 10^6^
*Stropharia caerulea*	1	2.5	1	1	8.6 ± 0.5	5.2 ± 0.3	1.5 × 10^5^
*Tubaria furfuracea*	3	4–4.8	1	3	7.5 ± 0.4	4.8 ± 0.2	8.8 ± 0.6 × 10^5^
*Tubaria hiemalis*	5	2.5–3.2	5	5	nd	nd	6.2 ± 1.4 × 10^4^
*Xerula* spec.	1	9.3	1	1	14.4 ± 0.7	10.9 ± 0.6	1.3 × 10^6^
Mushrooms with gill-like ridges or pseudolamellae							
*Hygrophoropsis aurantiaca*	1	5.5	1	1	5.7 ± 0.6	3.6 ± 0.4	2.4 × 10^5^
*Schizophyllum commune*	14	1.8-2.3	1	14	6.2 ± 0.3	2.2 ± 0.1	4.9 ± 2.0 × 10^6^
Mushrooms with pores							
Boletus luridus*	2	4.6-5.9	1/4	2	12.8 ± 0.5	5.6 ± 0.5	2.8 ± 1.9 × 10^5^
Boletus rhodoxanthus*	1	13.2	1/4	1	12.2 ± 0.5	4.7 ± 0.3	1.2 × 10^5^
Boletus splendidus*	1	15.3	1/4	1	12.7 ± 0.5	5.2 ± 0.4	3.7 × 10^5^
Chalciporus piperatus*	1	14.7	1/4	1	10.1 ± 0.5	3.5 ± 0.9	1.1 × 10^6^
*Laetiporus sulphureus*	1	5.8	1	1	5.8 ± 0.1	4.4 ± 0.4	7.8 × 10^5^
Suillellus queletii*	1	12	1/4	1	12.6 ± 0.5	6.4 ± 0.4	1.0 × 10^4^
*Suillus* spec.*	1	10.6	1/4	1	12.6 ± 0.5	5.5 ± 0.6	1.6 × 10^5^
Xerocomellus chrysenteron*	1	9.4	1/2	1	12.5 ± 0.7	5.3 ± 0.5	1.2 × 10^6^

^a^nd = not determined

^b^Averages of spore numbers included the data shown individually in Tables 1 and 3, the data for 20 h incubation of some mushroom species presented in Fig. 2a and b, and the 1 cm height values of mushrooms presented in Fig. 3 and the 18 h data for mushrooms presented in Fig. 6

### Spores and droplets in plastic lids

**Fig. 1:**
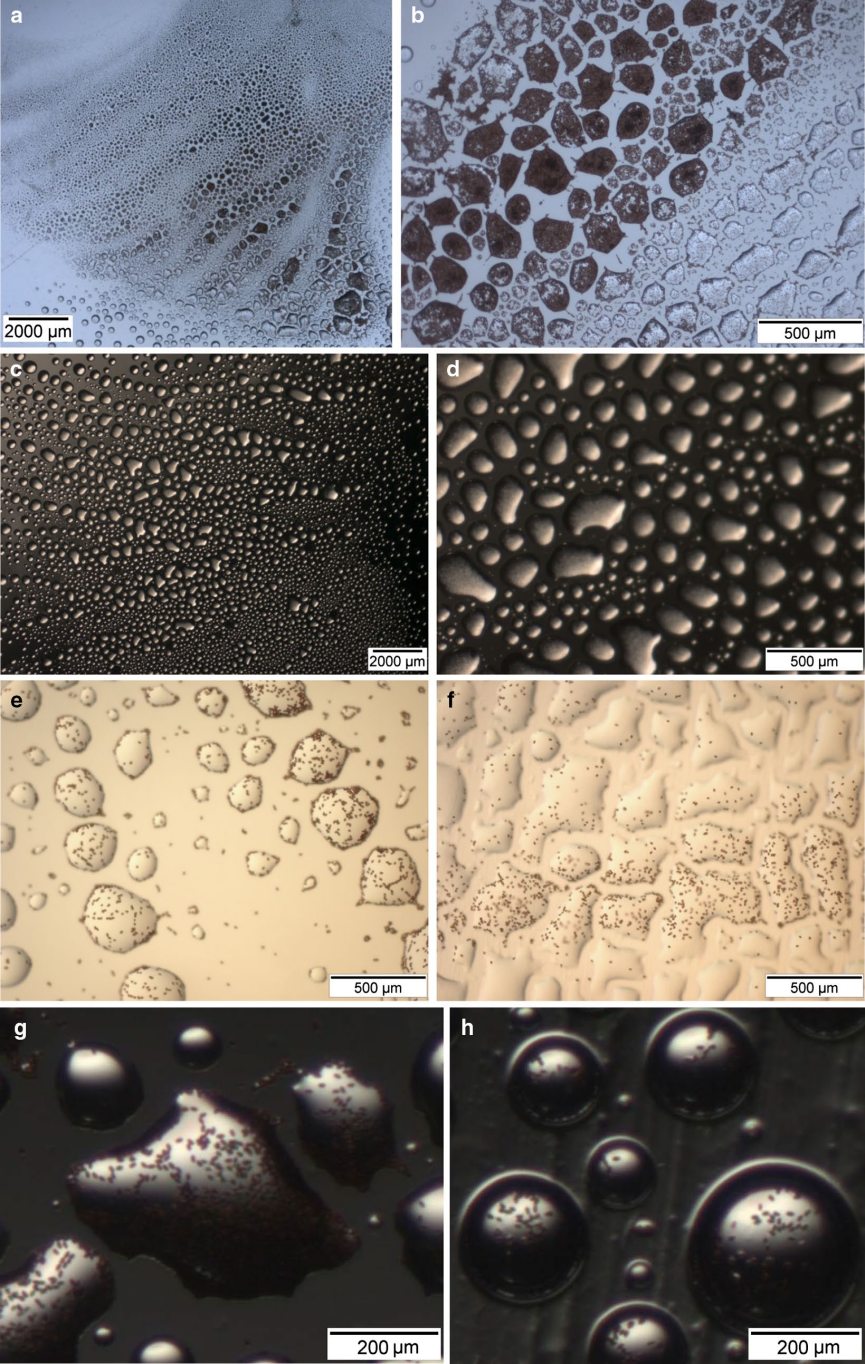
Basidiospores of *Coprinopsis domesticus* (**a**, **b**), *Schizophyllum commune* (**c**, **d**), *Coprinellus micaceus* (**e**, **f**), and *Psathyrella conopilus* (**g**, **h**) accumulated in droplets in lids of plastic Petri-dishes after 18 h incubation of full mushroom caps or halves of caps (*C. micaceus*) positioned upside-down on wet tissues in the base of the Petri-dishes. **a** and **c** show overviews on lamellar patterns recognized by the distribution of larger droplets with spores. Lamella positions are also reflected in the photos in **b**, **d**–**f** by the distinct accumulation of spores in the liquid droplets. The milky appearance of droplets in **d** is due to hyaline spores of *S. commune*. *Brown* spores of the other species can be recognized as dark spots in the droplet areas. Liquid droplets with spores spread irregular flat on untreated surfaces of plastic lids (**a**–**e**, **g**) while droplets of some species flattened more and those of others rounded more up when lids were covered with Vaseline (**f**, **h**)

Under standard incubation conditions (mushrooms laid upside-down on wet paper tissue in dishes covered with plastic lids), fine droplets usually developed overnight in the zones of the lids directly above the incubated reversed caps (Fig. 1a–d). Observations under the binocular revealed spores to be present in the droplets or, most common, spread at the surfaces of the droplets (Fig. 1e–h). Droplets with spores tended to spread somewhat irregularly flat over the plastic surfaces (Fig. 1) which suggests that they may contain some kind of surfactants. Regularly, patterns of lamellae were reflected in the lids due to the preferred positions and sizes of droplet formation. For species with dark spores, this was further visibly emphasized by their colour (Fig. 1a–f). The clear patterns of lamellae seen printed in the lids imply that spores fly straight up from their place of release toward the plastic lids where they collect with the growing droplets. Droplets however evaporated very fast upon opening of dishes. A clearly visible film of dried material was regularly left behind on the plastic surfaces (not further shown).

Spores were initially collected from the lids in 200 µl sterile water. Spores did however not easily transmit into the water but showed an affinity to stick to the plastic lid of a Petri-dish. Quick wiping with the plastic tip of a micro-pipette was required to transfer the spores into the liquid. Spores tended to clump and quickly sink to the bottoms of sterile Eppendorf cups into which spore solutions were transferred. Addition of mild detergent such as Tween 80 can help to suspend clumped basidiospores (Dhawale and Kessler 1993; Rincón et al. 2005). Therefore in later experiments, we used 200 µl sterile 0.1% Tween 80. With the detergent, spores were easily taken up from the lids and suspended.

### Species range with open hymenia tested

Caps or parts of caps of mushrooms of a broad taxonomic species range were incubated upside-down on wet paper tissue in dishes covered with plastic lids. In nearly all cases (i.e. for mushrooms of 66 distinct species), basidiospores collected in large numbers in the lids (Table 2). Fresh mushrooms with open hymenia were collected over the time of species with gills (in total 59 species from 12 different families of the *Agaricales*—i.e. 7 species from the *Agaricaceae*, 2 species from the *Amanitaceae*, 2 species from the *Bolbitiaceae*, 4 species from the *Inocybaceae*, 3 species from the *Hygrophoraceae*, 3 species from the *Marasmiaceae*, 1 species from the *Mycenaceae*, 2 species from the *Physalacriaceae*, 1 species from the *Plutaceae*, 25 species from the *Psathyrellaceae*, 5 species from the *Strophariaceae*, and 3 species from the *Tricholomataceae*; 1 species from the *Repetobasidiaceae*, *Hymenochaetales*; 1 species from the *Russulaceae*, *Russulales*), species with gill-like ridges (1 species from the *Hygrophoropsiceae*, *Boletales*) and pseudolamellae (1 species from the *Schizophyllaceae*, *Agaricales*), and species with pores (in total 7 species from the *Boletales*, i.e. 6 species from the *Boletaceae* and 1 species from the *Sulliaceae*; 1 species from the *Polyporaceae*, *Polyporales*). Nearly all tested species are characterized by four-spored basidia but *Agaricus bitorquis*, *Agaricus subperonatus*, *L. birnbaumii* and *Suillellus queletii* which can have both heterokaryotic and homokaryotic spores in their caps by mixed formation of bi- and four-spored basidia (Breitenbach and Kränzlin 1991, 1995). The only four exceptions of fleshy mushrooms which failed in spore collection in our experiments were single mature individuals of *Hygrocybe virginea* and *Hygrophorus eburneus* (both *Hygrophoraceae*), *Lepista saeva* (*Tricholomataceae*) and *Rickenella fibula* (*Repetobasidiaceae*). Since other species of the same or a closely related family gave spores in the lids (Table 2), these four failures did not relate to any specific taxonomic position of mushrooms but possibly to that spore shedding ended by fruiting body age (Haard and Kramer 1970; Li 2005; Saar and Parmasto 2014; not further analysed).

**Fig. 2:**
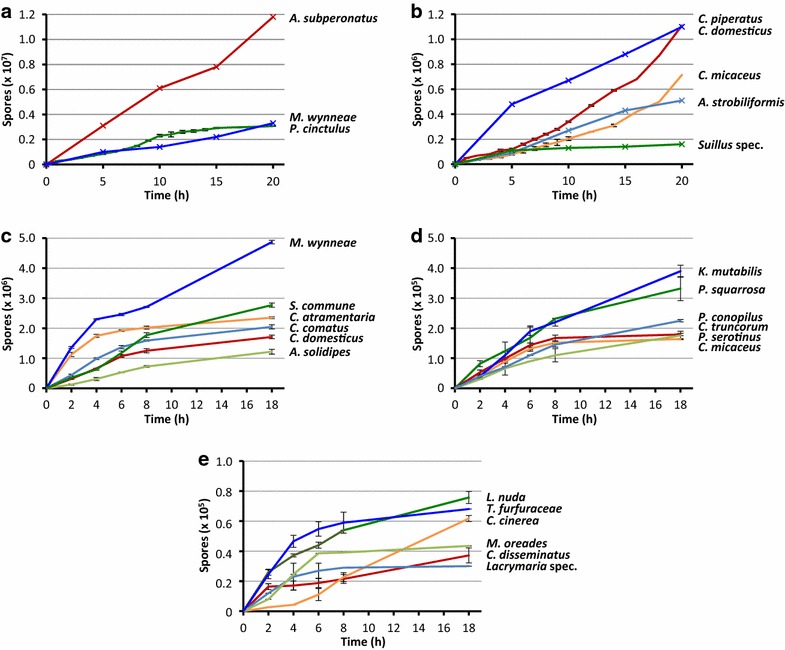
Accumulation of basidiospores in plastic lids over the time during incubation of mushrooms positioned upside-down on wet tissues in the base of Petri-dishes or glass jars. **a**, **b** Experimental Set-up 1: The two halves of different mushroom caps of *Paneolus cinctulus* (in total from 20 caps) and *Coprinellus micaceus* (in total from 15 caps) and whole caps of *Coprinellus domesticus* (in total 30 caps, all slightly dehydrated) were incubated in parallel in distinct Petri-dishes for fixed times to collect their spores in plastic lids, count the harvests and calculate from each two respective values the averages presented in the figures. Four complete caps were incubated in different dishes for *Marasmius wynneae*, the four quarters of single mushrooms for all other species. At distinct times (indicated in the figures by ×), the spores of an individual mushroom or an individual quarter were harvested for counting. **c**–**e** Experimental Set-up 2: Spores from whole fruiting bodies or parts of fruiting bodies were harvested at distinct time points and lids were then put back onto the Petri-dishes for further incubation and spore collection. Spores per point of harvest were counted and the different values for an individual sample were added together in order to calculate total spore numbers over the whole length of an incubation period. Average values with standard deviations were calculated for all species from each three distinct samples run in parallel. Halves of caps for *C. micaceus*, *Coprinellus truncorum*, *Coprinopsis atramentaria*, *Kuehneromyces mutabilis*, *Lepista nuda*, and *Panellus serotinus* were used, quarters of caps for *Pholiota squarrosa*, eighths of caps for *Coprinus comatus*, and complete mushrooms for all other species (for their full names see Table 2)

**Fig. 3:**
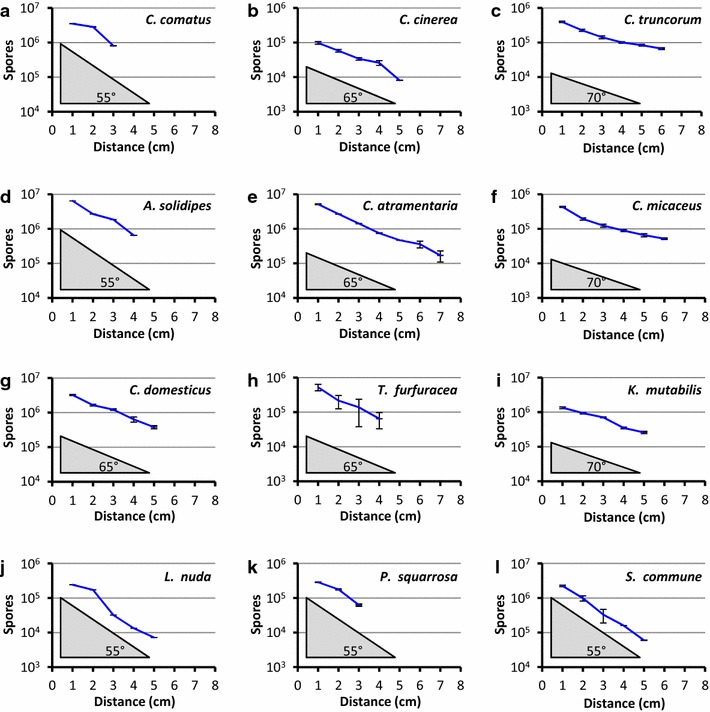
Accumulation of basidiospores in plastic lids positioned at different distances to the surfaces of mushrooms placed upside-down onto wet tissues in the base of Petri-dishes or glass jars. Spores were collected after 18 h incubation. Spore values were calculated as averages with standard deviations from each three distinct samples (*Kuehneromyces mutabilis*: each 1/2 of a mushroom; *Coprinopsis atramentaria*, *Lepista nuda*: each 1/4 of a mushroom; *Coprinus comatus*: each 1/8 of a mushroom; all others: complete mushrooms; for their full species names see Table 2). Values are shown for all distances where spores were found in the lids; lack of spores in lids (0 spores) after incubation is documented in the graphs by no value entry. In all instances, distances up to 10 cm were tested in 1-cm steps. Subfigures are arranged by spore lengths of the species, from longest (**a**) to shortest (**l**). **a**–**c**: 12.2–8.9 µm; **d**–**f**: 8.5–8.1 µm; **g**–**i**: 7.6–7.3 µm; **j**–**l**: 6.8–6.2 µm (for detailed data compare the entries in Table 2). *Grey shaded triangles* with angle values given for the right corner (55°, 65° or 70°) indicate grouping by similar steepness of logarithmic decrease in spore numbers with increasing distance to the lids

Spore solutions from mushrooms incubated upside-down overnight in Petri-dishes typically contained between about 10^4^ up to in highest cases >10^7^ total spores (compare Table 2). Spore yields obtained from different mushroom samples of a same species were usually very similar, even when harvested and incubated at different days (details not further shown but see the averaged data in Table 2 and compare the data for individual species also with those from specific experiments presented in Tables 1, 3; Figs. 2, 3). This suggests in coincidence with earlier reports on spore releases of different species (Fischer and Money 2009; Saar and Salm 2014) that species-specific parameters determine the frequency of spore release and the actual spore capture in the plastic lids. However, spore yields did not plainly depend on a single simple parameter such as cap size, spore sizes, structures of hymenia (lamellae, ridges or pores) (Table 2), or gill numbers per cap and pore diameters (see the cited field guides for the individual parameters of species for comparison with the spore collection data in Table 2). Cap age and speed of younger cap maturation, lengths and mode (consecutive or synchronous) of spore production and maturation periods (Kües and Navarro-Gonzaléz 2015; Halbwachs and Bässler 2015) might also be needed to be considered on individual species level as parameters of potential influence on spore harvests.

**Table 3:** Spore harvests in lids after 18 h incubation of mushrooms in upside-down position in Petri-dishes on wet paper tissues

Species	No of mushrooms per situation	Part used	No of samples	Spore harvests			
				Plastic lid		Glass lid	
				Plain	With Vaseline	Plain	With Vaseline
*Coprinellus micaceus*	2	1/2	3	5.1 ± 3.0 × 10^5^	2.1 ± 1.0 × 10^5^	6.0 ± 3.3	7.3 ± 5.3
*Coprinellus domesticus*	3	1	3	4.9 ± 1.4 × 10^6^	1.6 ± 0.5 × 10^6^	11.0 ± 6.5	20.0 ± 13.1
*Coprinellus disseminatus*	5	1	5	2.1 ± 0.9 × 10^5^	4.5 ± 1.1 × 10^5^	11.7 ± 9.4	24.0 ± 19.6
*Coprinopsis atramentaria*	1	1/2	2	5.9 ± 0.4 × 10^6^	4.1 ± 0.4 × 10^6^	0	0
*Coprinus comatus*	1	1/8	2	9.2 ± 0.4 × 10^6^	5.7 ± 0.5 × 10^6^	0	0
*Hypholoma fasciculare*	3	1	3	1.0 ± 0.5 × 10^5^	0.9 ± 0.5 × 10^5^	0	0
*Psathyrella candolleana*	1	1	1	9.4 × 10^5^	7.6 × 10^5^	0	0

### Effects of mushroom conditions on spore harvests

We collected mostly fresh fleshy mushrooms of Agarics (Table 2) and many were still young, in the stage of opening or close to be fully opened. During overnight incubation in reversed orientation, younger caps of most of the fleshy species further opened and cap diameters (measured at the time of harvest from nature, see Table 2) tended to further extend by stretching out the umbrellas. Of the lamellate species, the single fruiting bodies of *Agaricus campestris*, *Agaricus subfloccosus*, *Hygrophorus olivaceoalbus*, *Inocybe fraudans*, *Marasmius oreades*, and *Psathyrella spadiceogrisea*, the two mushrooms of *Pluteus* spec., all four of *Marasmius wynnei* and all six of *Psathyrella candolleana* were already fully open at their harvest. There were no problems with any of the younger and the here listed mature mushrooms to obtain high numbers of spores transferred to the plastic lids positioned above (Table 2).

Notably, also younger (*P. conopilus*), mature (the single fruiting bodies of *Agaricus augustus*, *Pholiota* spec. and *Stropharia caerulea*; one mushroom each of *P. candolleana* and *S. commune*, two mushrooms each of *Panaeolus ater* and *Psathyrella atrolaminata*, and a series of mushrooms of *C. domesticus*) or aging mushrooms (*Conocybe tenera*, one mushroom each of *Agrocybe dura*, *Marasmius cohaerens* and *Panaeolus cinctulus*) which were to different extend desiccated were successfully appointed in collecting spores in numbers of 10^4^–10^6^ in plastic lids by reversed incubation on wet paper tissue (Table 2; Fig. 2b). The shrivelled mushrooms refreshed in shape by taking up humidity from the wet paper tissue. For *A. dura*, we had an older dry fruiting body and young fresh mushrooms. Spore harvests were similar (2.6 × 10^6^ spores versus from 2.0 to 3.5 × 10^6^ spores). Spore numbers for dehydrated mushrooms of *C. domesticus* were only somewhat reduced (0.9 ± 0.1 × 10^6^; Fig. 2b) as compared to most mushrooms harvested in fresh stage (4.1 ± 1.8 1 × 10^6^; Table 2). Moreover, mushrooms of the durable species *S. commune* which were dried on purpose at RT for 15 days gave still considerable numbers of spores in lids (5.6 ± 0.8 × 10^4^; n = 3; tested with the water agar system) over the 18 h of reversed incubation although these were 100× reduced as compared to mushrooms which were used directly at the day of harvest (Table 1). *S. commune* in active phases produces continuously new spores (Kües and Navarro-Gonzaléz 2015) by which fresh mushrooms might distinguish from revived specimens that will require time for full physiological recovery.

**Fig. 4:**
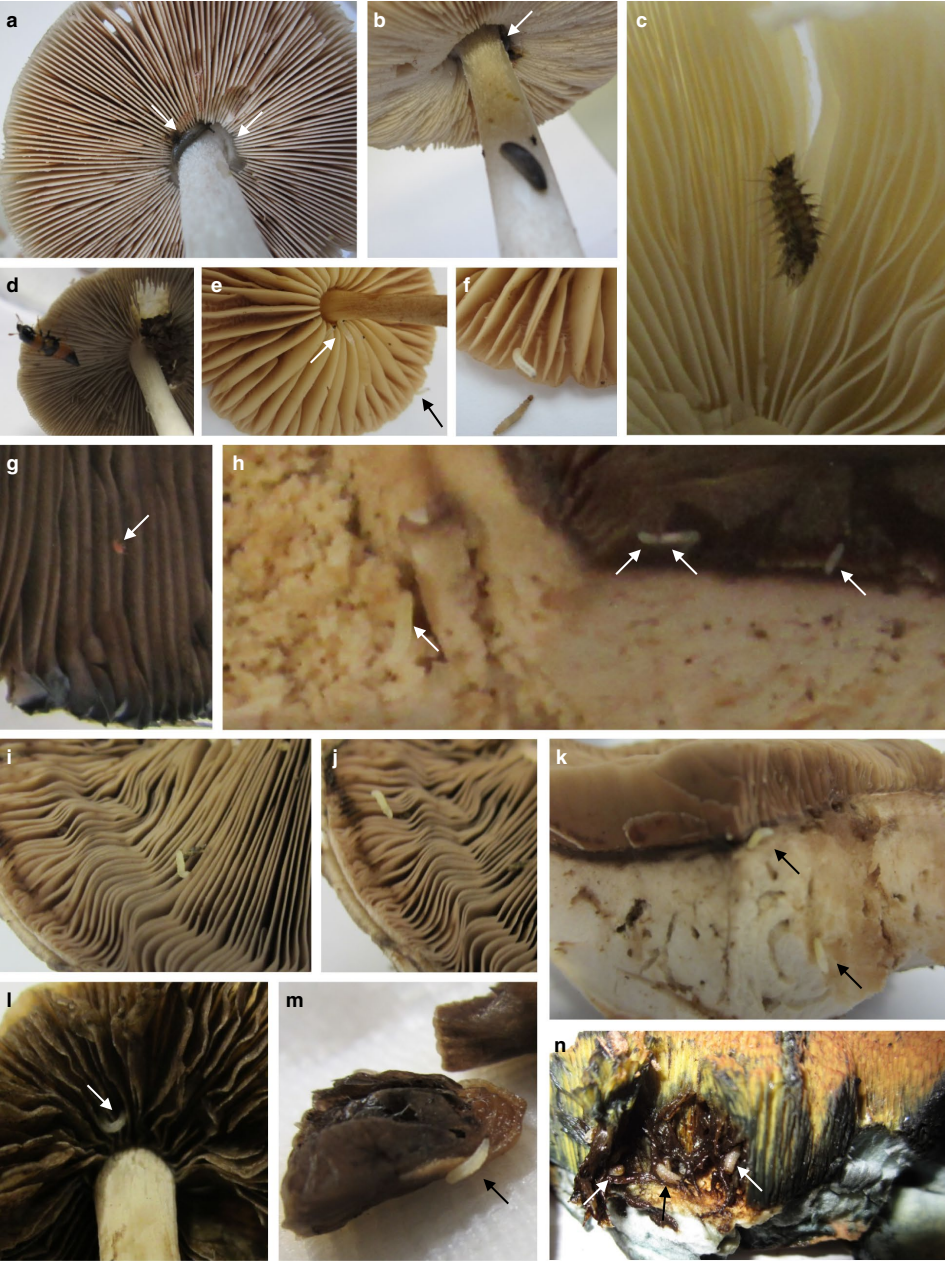
Examples of mushrooms infested by small animals. **a**, **b** *Pluteus* spec. mushroom with small slugs. Different insect larvae were found on mushrooms of **c** *Pholiotina vestita*, **e**, **f** *Marasmius wynneae* (one of four animal-infested mushrooms tested for sporulation is shown), **h** *Agaricus campestris*, **i**–**k** *Agaricus subperonatus*, **l**, **m**, *Paneolus cinctulus* (one of in total six mushrooms tested for sporulation was visibly animal-infested), and **n** *Suillellus queletii* (note the still intact pores and tubes at the upper area of the photo). **d** An adult beetle of *Oxyporus rufus* (*Coleoptera*, *Polyphaga*) on *Psathyrella spadiceogrisea*. **g** *A. campestris* with red mites (same mushroom as in **h**). *White* and *black arrows* point to positions in the photos of animals less easy to detect. All mushrooms shown in the figure were used in reversed position on wet tissues to collect spores in plastic lids above and all gave high numbers of spores (data are included in Table 2)

Further of importance, also mushrooms infested with small animals (Fig. 4) can be used in collecting spores in lids. Of the lamellate mushrooms mentioned above, *Pluteus* spec. for example carried small slugs (Fig. 4a, b), on *P. spadiceogrisea* and *A. campestris* were rove beetles (Fig. 4d) and red mites and internally some larvae (Fig. 4g, h), while the ones of *A. campestris* (Fig. 4h), *A. subperonatus* (Fig. 4i–k) and *M. wynneae* (Fig. 4e, f), one of six of *P. cinctulus* (Fig. 4l, m) and one of two mushrooms of *Pholiotina vestita* (Fig. 4c) had grubs. Particularly the fleshy pilei of the *A. campestris* (Fig. 4h) and *A. subperonatus* fruiting bodies (Fig. 4i–k) and also all mushrooms of the *Boletales* (Fig. 4n and not shown) were much infested with many grubs. Insect larvae had eaten tunnels into the pilei of the *S. queletii* (Fig. 4n) and the other *Boletales*’ mushrooms. However, there was still much surface area with intact pores (Fig. 4n and not shown) in order to obtain bulks of basidiospores in plastic lids above (Table 2). Only for an aged, slug-eroded and fully grub-populated decaying *Xerocomellus chrysenteron* fruiting body, it was not possible to obtain spores from. Where we had infested and animal-free fruiting bodies, harvested spore numbers were still similar (infested decaying *C. domesticus* fruiting body: 0.9 × 10^6^ spores, animal-free mushrooms: from 1.3 to 6.9 × 10^6^ spores; infested *P. cinctulus* fruiting body: 0.9 × 10^6^ spores, animal-free mushrooms: from 2.9 to 5.4 × 10^6^ spores).

### Spore release over the time

Mushrooms started quickly to propel off spores when incubated upside-down on wet tissues in closed dishes (Fig. 2). Already after 2 h of incubation, considerable amounts of spores (>10^4^–10^5^ for most species or for somes species even 10^6^) could be harvested from mature fruiting bodies in plastic lids. Fast spore release continued for a few hours (4–6 h) but the speed of spore release usually decreased for several of the species with time of incubation to eventually level off to a maximum amount of spores which possibly can be discharged by a single specimen under the experimental conditions applied.

In many cases, we used in our experiments fleshy mushrooms of limited life-time such as of the ephemeral inkcaps (species of *Coprinellus*, *Coprinopsis*, *Coprinus*) that after a transitory period of ballistospore ejection release a majority of their spores in liquid droplets by autolysing their caps (McLaughlin et al. 1985; Kües 2000; Redhead et al. 2001; Nagy et al. 2013). The time of spore release by ballistospore ejection up to onset of cap autolysis was always sufficient to obtain high spore numbers in lids of plastic Petri-dishes, in amounts of >10^5^ to >10^6^ (Tables 1, 2, 3; Figs. 2, 3). In case of *C. cinerea* fruiting bodies from laboratory cultures, spore release was delayed by 4 h from the start of incubation of young opening caps with still pale gills (Fig. 2e). This time coincided well with the known time schedule of basidiospore maturation with corresponding black gill staining after light-induced synchronized karyogamy in the basidia (Kües and Navarro-Gonzaléz 2015).

### Spore attachment to lids of different material

In preliminary experiments, mushrooms of *S. commune* and of *C. atramentaria* were incubated upside-down in dishes with plastic lids or in dishes with glass lids. Spore collected overnight in plastic lids whereas spores were not present in glass lids (data not shown). The results suggested that an electrostatic disposition possibly helped to attach ejected spores to the surface of the plastic lids.

We smeared sticky Vaseline onto glass and plastic lids of Petri-dishes to support attachment of spores to the surfaces. Experiments with parallel sets of overnight upside-down incubated mushrooms or parts of mushrooms of *Hypholoma fasciculare*, *P. candolleana* and five different inkcap species showed that Vaseline in plastic lids had no incisive negative effect on spore yields (Table 3; Fig. 1e–h). Spores stuck well in droplets to the Vaseline (Fig. 1f, h) although it was harder to harvest them from Vaseline and bring them into solution than without. However, Vaseline did not lead to considerably increased numbers of spores that attached to glass lids. With and without Vaseline, there were always either no or only negligible few spores in glass lids (Table 3). We conclude from the experiments that different affinities between the glass and plastic surfaces of tying spores is not the primary cause for the unequal spore harvests from lids of different material but a distinct effect of the plastic on upward spore transfer. Likely, static electricity of the plastic (Woodland and Ziegler 1951; Kuo 2015) will force spores to fly upward onto the lids where the same forces will then support attachment of the spores to the plastic surface.

### Spore transfer to plastic lids over different distances

The data in Table 1 implicate for several species that not all basidiospores released from reversed mushrooms are attracted to plastic lids. Experiments in which the distance between the upside-down laid mushrooms and the plastic lids above for several species were varied further support an influence by spore properties on spore yields in the lids. For all species, numbers of spores attached to the lids decreased in linear trends on the log scale with increasing distance to the reversed mushrooms (Fig. 3). Roughly, three groups in line steepness might be distinguished (Fig. 3a, d, j–l, steepest: *C. comatus*, *A. solidipes*, *Lepista nuda*, *P. squarrosa*, *S. commune*; Fig. 3b, e, g, h, medium: *C. cinerea*, *C. atramentaria*, *C. domesticus*, *Tubaria furfuracea*; Fig. 3c, f, i, flattest: *C. truncorum*, *C. micaceus*, *Kuehneromyces mutabilis*). Break-offs of all lines (lowest distances where no spores detected in lids) were abrupt. Between species, break-offs occurred at different heights, following different orders of magnitudes (ranging from <10^4^ up to nearly 10^6^) of absolute spore numbers at the last positive height. Absolute numbers of spores collected in lids at low distances did not correspond throughout to the possible longest distance over which spores of a species were found to be attracted to plastic lids (Fig. 3). *C. atramentaria* as a species with highest spore numbers in lids at short distance (>10^7^ at 1 cm distance) also yielded reasonable amounts of spores in lids at highest distances (>10^5^ at 7 cm distance; Fig. 3e), followed by *Coprinellus truncorum* and *C. micaceus* with both ca. 4 × 10^5^ spores in lids at 1 cm distance and ca. 5 to 7 × 10^4^ spores at 6 cm distance (Fig. 3c, f). In all other cases, maximum heights of spore detection varied between 3 cm and 5 cm distance. Spore sizes (lengths, see Table 1) or volumes (as calculated for an ellipsoid V = 4/3 π × ½ length × 2 × ½ width; data not shown) did also not correlate with maximum heights at which spores were attracted to plastic lids (Fig. 3). Attraction between higher up-flying spores and the plastic lids might be expected to be stronger than between less far up-flying spores and the lids. Basidiospores do also have electric charges (Buller 1909, 1922; Gregory 1957; Swinbank et al. 1964; Webster et al. 1988; Saar 2013; Saar and Parmasto 2014; Saar and Salm 2014). Individual differences in charging of spores remain as explanation for the observations.

### Capturing spores from gasteroid basidiomycetes

We concluded before that spore collection in plastic lids positioned above mushrooms with open hymenium depended on their forcible ballistospore discharge mechanism mediated at high humidity by the fusion of Buller’s drops with liquid films at the adaxial sides of the spores. Gasteroid species have lost this active basidiospore discharge mechanism, produce their basidiospores within closed fruiting bodies and disperse them from openings or cracks passively with wind or through pushing the spore sacs by rain drops (Hibbett et al. 1997; Wilson et al. 2011; Kües and Navarro-Gonzaléz 2015). Accordingly, basidiospores did not accumulate during overnight windstill incubation in evaporating dishes (18–24 h) in the plastic lids above mature puffballs (*Lycoperdon perlatum*, *Agaricaceae*) which were either already cracked or of which the inner gleba with the basidiospores was opened by cutting the fruiting body into two equal halves. Similarly, there were no spores in lids after 18 h incubation when we positioned two different mature earth-stars (*Geastrum rufescens* and *Geastrum striatum*; *Geastraceae*, *Geastrales*) with an ostiole as a natural opening and unrolled segments of the exoperidium into windstill evaporating dishes covered by plastic lids. However, when we hit 2× with caution the spore sacs from the side with a glass rod through the spouts of the evaporating dishes, clouds of spores escaped through the ostioles from the spore sacs into the air and attached to the plastic lids above (not further shown). We harvested and counted in the lids then 6.9 × 10^6^ basidiospores of *G. rufescens* (plating proofed them to be contamination free) and 4.4 × 10^6^ for *G. striatum*. We conclude that the spores are moved into the air is decisive for spore attraction to the plastic lids but not the particular initial mode of the spore release.

### Spore plating

**Fig. 5:**
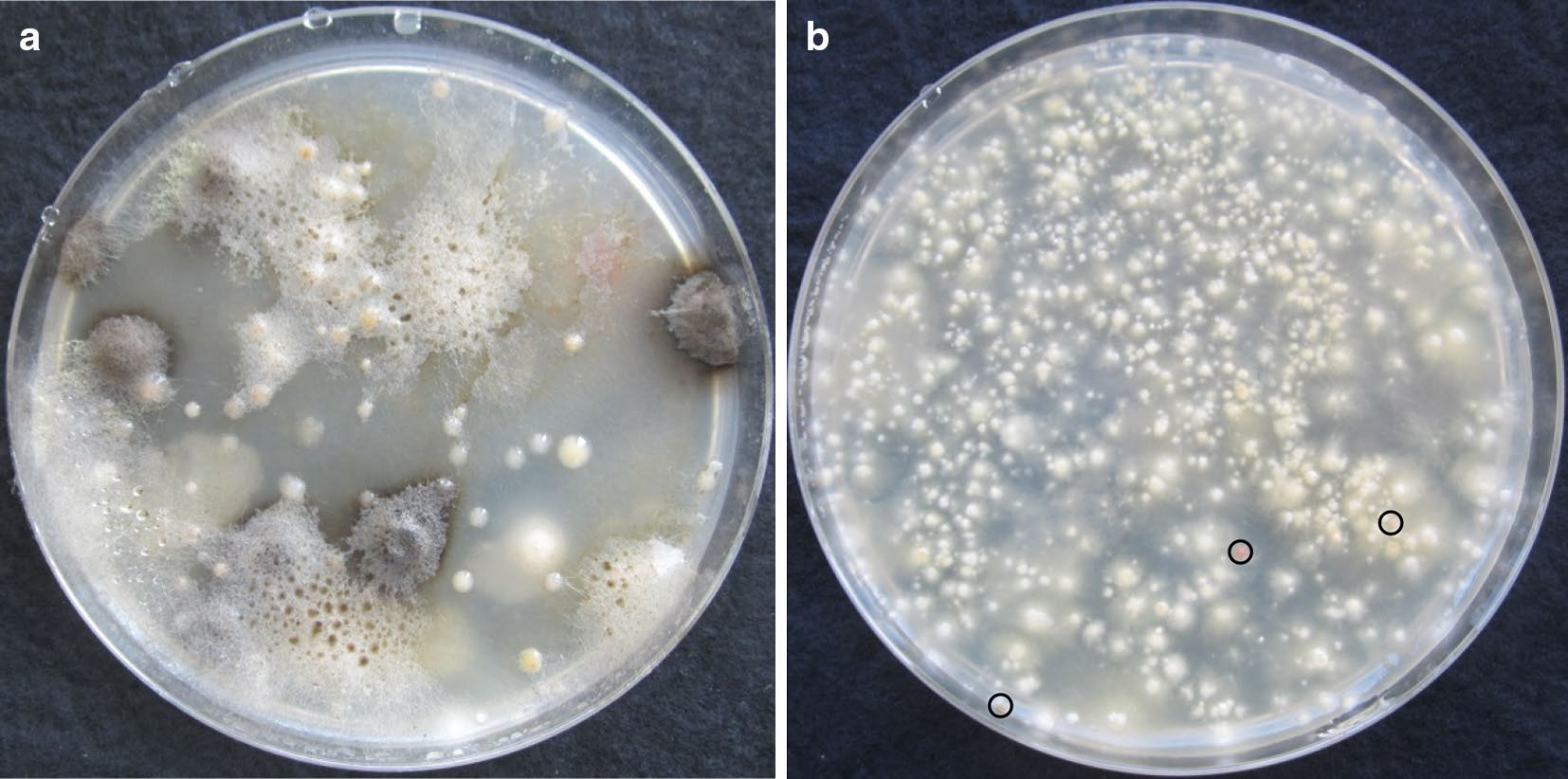
Outgrowth of microbes from 50 µl basidiospore solutions of *Tubaria hiemalis* on MEA + AB during incubation at 25 °C. **a** Spore solution from a classic spore print (from the 2nd mushroom incubated in upside-top condition in Table 4). Different species of molds have overgrown the *T. hiemalis* basidiospores as well as colonies of different yeast species. **b** Spore solution from a spore print collected in the plastic lid of a Petri-dish (from the 2nd mushroom incubated in upside-down condition in Table 4). Colonies of 2437 germinated basidiospores are seen (27% germination rate). Encircled are three red yeast colonies found as the only contaminations grown on the plate

The reliable yields of spores from upside-down positioned wild mushrooms in the plastic lids of Petri-dishes evoked the further idea to test whether just basidiospores were released onto the lids. First, 50 µl aliquots of spore solutions from individual mushrooms from the Experimental Set-up 1 in Table 1 were analysed on MEA media. Spore solutions from mushrooms incubated in Petri-dishes in natural direction gave rise to massive bacterial and also numerous fungal contaminations, in contrast to aliquots from spore suspensions which were harvested from plastic lids from upside-down incubations of mushrooms (Table 4). Generally, sizeable bacterial colonies appeared on media after 1 day at 25 °C incubation, colonies of yeasts and molds after 2 days and basidiospore germlings visible to the naked eye for all tested mushrooms after 3 days, respectively. Both bacterial and fungal contaminants (molds, mostly *Ascomycetes*) hindered by overgrowth other organisms in growth, including germination of the basidiospores and growth of germlings of the respective mushroom plated (Table 4). Addition of antibiotics suppressed bacterial growth but fast growing molds present in the samples of classic spore prints still overgrow the basidiospores (Table 4; Fig. 5a). Colonies from germinated basidiospores of spore suspensions from classical prints among contaminations were therefore only observed in the cases of *C. domesticus* and *S. commune* but at much lower frequency (≥1000 fold less) than on plates onto which samples of basidiospores were plated from harvests from plastic lids located above upside-down incubated mushrooms (Table 4). In absence of contaminants, germination of basidiospores from samples harvested from plastic lids was unhindered for all mushrooms of the four species tested. There were no bacterial contaminations and fungal contamination occurred only in one instance (=10% of all tested cases) with just 3 yeast colonies (Table 4; Fig. 5b). While the germination rates of basidiospores were different between the species used (Table 4), possible effects by densities of the own basidiospores in the solutions were not further tested.

**Table 4:** Contaminations of basidiospore solutions (from Experimental set-up 1 in Table 1) with other microbes

Fruiting body	Classic spore print (mushroom upside-top)						
	Basidiospores in 50 µl	Colonies on plate/50 µl spore solution plated					
		MEA			MEA + AB		
		Basidiomycete	Contamination^a^		Basidiomycete	Contamination^a^	
			Bacteria	Other fungi		Bacteria	Other fungi
*Coprinellus domesticus*	1.6 × 10^7^	68	Uncountable	2 yeasts, 34 molds	51	–	122 yeasts, 14 molds
*Panaeolus cinctulus*	8.0 × 10^6^	–	5.4 × 10^3^	3 yeasts, 38 molds	–	–	8 yeasts, 14 molds
*Schizophyllum commune*	2.1 × 10^7^	2563	Uncountable	34 yeasts, 4 molds	2326	–	544 yeasts, 8 molds
*Tubaria hiemalis* (1st mushroom)	1.9 × 10^4^	–	1.7 × 10^5^	14 yeasts, 6 molds	–	–	42 yeasts, 10 molds
*T. hiemalis* (2nd mushroom)	2.4 × 10^4^	–	4.3 × 10^5^	6 yeasts, 5 molds	–	–	64 yeasts, 21 molds

	Spore print in plastic lid (mushroom upside-down)						
	Basidiospores in 50 µl	Colonies in plate/50 µl spore solution plated					
		MEA			MEA + AB		
		Basidiomycete	Contamination		Basidiomycete	Contamination	
			Bacteria	Other fungi		Bacteria	Fungi
*C. domesticus*	1.2 × 10^6^	4660 (0.33% germination)	–	–	4852 (0.34% germination)	–	–
*P. cinctulus*	9.0 × 10^5^	1352 (0.15% germination)	–	–	1280 (0.14% germination)	–	–
*S. commune*	1.8 × 10^6^	Uncountable	–	–	Uncountable	–	–
*T. hiemalis* (1st mushroom)	7.8 × 10^3^	2458 (31.5% germination)	–	–	2873 (36.8% germination)	–	–
*T. hiemalis* (2nd mushroom)	9.0 × 10^3^	2213 (24.5% germination)	–	–	2437 (27.0% germination)	–	3 yeasts

^a^Numbers of fungi in different media did not clearly correspond to each other since fast bacterial growth suppressed fungal growth and fast growing molds suppressed that of other molds and yeasts

Second, spore suspensions from classical prints of *C. domesticus* and *P. papilionaceus* mushrooms fixed to dishes by agar during downward spore shedding were compared on LB medium at 25 °C with spore suspensions obtained by harvests from plastic lids from agar-fixed mushrooms incubated upside-down (solutions from the Experimental Set-up 2 in Table 1). In case of the classical *C. domesticus* spore print (5.3 × 10^6^ spores/50 µl), >360 large slimy and blurred bacterial colonies grew over the surface of the plate and five yeast colonies on medium with antibiotics while the plates with spore suspensions from the upside-down incubated fruiting body (6.0 × 10^5^ spores/50 µl) were all free of any contaminations (but also of germinated basidiospores; not further shown). Judging by colony morphologies, multiple bacterial species (>10) grew in high density of a plated basidiospore suspension from the classical spore print of *P. papilionaceus* (8.8 × 10^5^ spores/50 µl) and still three types of bacteria (338, 15 and 3 colonies, respectively) when antibiotics were added to the growth medium. This contrasted the situation with the spore solution from the upside-down incubated mushroom (1.0 × 10^5^ spores/50 µl) where no bacteria were found on LB without or with antibiotics while germination of basidiospores at 0.72% frequency was observed (not further shown).

**Fig. 6:**
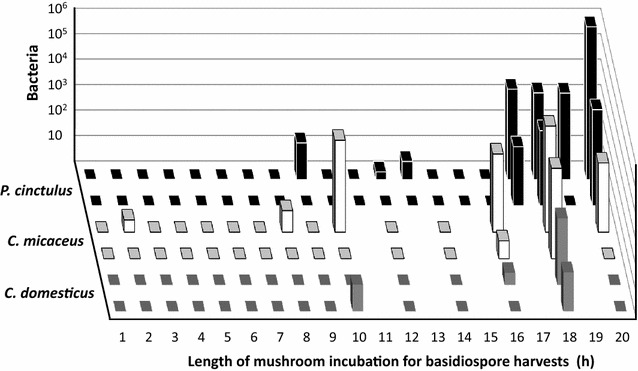
Bacterial contaminations in spore solutions of *Paneolus cinctulus* (*black columns*), *Coprinellus micaceus* (*white columns*), and *Coprinellus domesticus* mushrooms (*grey columns*). After upside-down incubation of mushrooms for basidiospore harvests from plastic lids (Fig. 2a, b), each 50 µl of spore solutions were plated onto MEA medium, incubated at 25 °C for 3 days and bacterial colonies were counted. Two different basidiospore solutions were always tested per time point for a species. For *P. cinctulus* and *C. micaceus*, these came always from the two halves of a same mushroom which were incubated for spore collection in parallel in different Petri-dishes. For *C. domesticus*, the each two parallel spore collections came from different mushrooms. Bacterial colonies in the *P. cinctulus* sample B, 20 h (shown in the figure at furthest distance to the observer) were uncountable why 10^6^ bacteria was used in the figure as an arbitrary estimate

From the time course experiment presented in Fig. 2, we had each two samples for *C. domesticus* (Fig. 2b), *C. micaceus* (Fig. 2b) and *P. cinctulus* (Fig. 2a) per tested time point of spore release into lids of plastic Petri-dishes. We tested also these for presence of contaminations by cultivation on MEA at 25 °C. In case of *C. domesticus* 26 of 30 basidiospore samples tested (87%) were free of contaminants, in case of *C. micaceus* 22 of 30 samples tested (73%), and in case of *P. cinctulus* 30 of 40 samples tested (75%). When contaminants were observed (in 22% of all these cases together), these were always bacteria (usually 1 or 2 types, rarely 3 types of bacteria) while the number of bacteria per 50 µl plated spore suspensions differed between 3 and 407 for *C. domesticus*, 3 and 14,400 for *C. micaceus* and between 2 and >10^6^ for *P. cinctulus* (Fig. 6). There was a tendency in likelihood for samples obtained from longer mushroom incubation to contain contaminations but there was no continuous increase in bacterial numbers over the different samples with length of mushroom incubation applied for spore release. Moreover, samples from two different halves of a same mushroom often differed in that bacteria were observed in only one of the two (Fig. 6). Furthermore, spore samples (50 µl) of the each four analysed time periods (5, 10, 15, 20 h) of spore release of *A. strobiliformis* (Fig. 2b), *A. subperonatus* (Fig. 2a), *C. piperatus* (Fig. 2b), *M. wynneae* (Fig. 2a), and *Suillus* spec. (Fig. 2b) were all free of bacteria but the 15 h sample of *C. piperatus* (1 bacterial colony), the 20 h sample of *M. wynneae* (1124 bacterial colonies) and the 20 h sample of *Suillus* spec. (2 yeast colonies). Thus, also in these series of samples, when contaminations were found (in 15% of all cases; 10% bacterial contaminations; 5% fungal contaminations) they came from longer mushroom incubations appointed for spore shedding.

Finally, spore solutions (50 µl) from 18 or 20 h incubation of mushrooms in upside-down position of a broader range of 19 randomly collected species (from Table 2) were tested on MEA. No contaminants were found in spore suspensions of individual fruiting bodies or of parts of fruiting bodies of *A. augustus*, *A. bitorquis*, *A. campestris* (grub infested; Fig. 4g, h), *A. subfloccosus*, *A. subperonatus* (grub infested; Fig. 4i–k), *Amanita excelsa*, *Hygrocybe conica*, *L. sulphureus*, *L. birnbaumii*, *Pholiota* spec., *P. vestita* (of two tested, one was grub infested; Fig. 4c), *Psathyrella spadiceogrisea* (with beetle; Fig. 4d), and *X. chrysenteron* (grub infested). Contaminations were found in one of two tested *A. dura* spore solutions (203 colonies, 1 type of bacteria), in one of the solutions from two tested grub-infested *Boletus luridus* fruiting bodies (20 colonies of yeasts), and in spore solutions of *H. olivaceoalbus* (59 bacteria, 1 type), *Inocybe erubescens* (3 yeast colonies), and grub-infested mushrooms of *Boletus rhodoxanthus* (312 colonies, 2 types of bacteria) and *S. queletii* (2128 colonies, 3 types of bacteria); i.e., contaminations in spore solutions in this series of platings were discovered in solutions of 27.3% of all tested mushrooms (i.e. in 20.7% of all individual samples tested) with 18.2% (13.8%) bacterial infections and 9.1% (6.9%) fungal contaminations. As a further important aspect, it should be recalled that in total 9 of these 22 mushrooms tested (Fig. 4d and not further shown) were infested with animals while only 3 spore solutions of these were contaminated.

Considering all plating test series together, 19 mushrooms infested with animals were analysed (from 15 different species; 28 different samples). Spore solutions of only 7 of these (25.0% in total; 17.9% with bacteria, 7.1% with fungi) were found contaminated.

## Discussion

Classically, when spore prints are to be produced from mushrooms with open hymenia such as for species identification or when individual basidiospores are to be isolated for germination, mushroom caps are laid facing down onto a surface (see “Introduction” section), in compliance to their orientation in nature. Under saturating levels of humidity, matured basidiospores are propelled off from the sterigmata on their basidia by fast hygroscopic development and subsequent actions of Buller’s drop. The surface energy obtained from the Buller’s drop by its fusion with the also hygroscopic liquid adaxial spore film is calculated to be sufficient for the spores to just reach the middle in between two lamellae or ridges or of a pore in order to then fall down out of the mushroom by gravity (Ingold 1939, 1957, 1992; Webster et al. 1984a, 1989; Turner and Webster 1991; Pringle et al. 2005; Noblin et al. 2009; Fischer et al. 2010a, b). In this study, we show indirectly by a dependence on high humidity that ejection of ballistospores from their sterigmata is a prerequisite for later collection of basidiospores from plastic lids positioned above upside-down incubated mushrooms with open hymenia. This is however not all.

### Driving forces for spore flight

If only the catapulting energy and gravity will be the acting driving forces for the spores to move upon release, accumulation of basidiospores against gravity in the lids of plastic Petri-dishes would not be possible. The energy of catapulted basidiospores is quickly used up during the propelling into the free airspace by the braking effect through the viscosity of the air (Turner and Webster 1991; Pringle et al. 2005; Stolze-Rybczynski et al. 2009; Fischer et al. 2010a, b). Once fully braked, the spores should then drift downward by action of gravity, which in case of upside-down positioned mushrooms would be down into the free gaps between hymenia. This reasonable prospect made us wondering what in our experiments might be the reason for about 10% to up to 100% of the basidiospores from a mushroom to instead fly up and attach to the plastic Petri-dish lids (Table 1).

The air space in the closed Petri-dishes can be expected to be motionless. Concerted release of basidiospores by a mushroom and evaporate cooling of the air surrounding the cap can principally help that the spores are whirled up in clouds by created airflows (Buller 1934; Deering et al. 2001; Dressaire et al. 2015, 2016). However, the clear lamellar patterns of mushrooms reproduced in our experiments by the spore repositories in the plastic lids speak against any generation of influential eddies in the closer head space of the mushrooms. Our investigations revealed that electrostatic charges by plastic are the likely reason for spores to move straight up against gravity and also for them to attach to the attracting plastic (Table 1). However, charges innate to the spores should also have their part in this process (Fig. 3). Basidiospores when ballistically discarded from mushrooms are indeed electrically charged. Charges of endogenous origin are possibly connected to the mass transfer when Buller’s drop suddenly fuses with the liquid film on the adaxial side of a spore. In addition, airflow will create charges on spores by triboelectric effects (Buller 1909; Webster et al. 1988; Saar and Parmasto 2014; Saar and Salm 2014) which could give an extra stimulus to the spores to leave the caps against gravity by electrostatic attraction to plastic lids.

In species-specific manner, spores of an individual mushroom can differ in type of charge (+ or −) and in strength of charging. The charges cause in horizontal electric fields that spores shed from mushrooms will drift away to one side. In many species, there are populations of positively and of negatively charged spores, while spores in some species are unipolar-positively and in some others unipolar-negatively charged (Buller 1922; Gregory 1957; Webster et al. 1988; Saar 2013; Saar and Parmasto 2014; Saar and Salm 2014). Of the species that we tested in this study (Table 2), absolute charges of basidiospores have been estimated before for *A. campestris* in different experimental series between 1.55/0.39/0.39 and 3.10/3.11/2.16 × 10^−17^ C (mean 2.23/1.28/0.93 × 10^−17^ C), for *C. micaceus* between 0.85 and 4.22 × 10^−17^ C (mean 2.14 × 10^−17^ C), for *L. nuda* 1.88 × 10^−17^ C, for *M. oreades* between 1.25 and 10.12 × 10^−17^ C (mean 4.37 × 10^−17^ C), for two *Pholiota* spec. strains between 1.65 and 2.03 × 10^−17^ C, and for *S. commune* between 1.70 and 9.55 × 10^−17^ C (mean 4.10 × 10^−17^ C), (Webster et al. 1988; Saar and Salm 2014). Populations of basidiospores of *L. nuda* were found to be 97% negatively charged, whereas populations of *A. campestris* and *Pholiota* species were bipolar with more numerous positively charged spores and populations of *C. micaceus* bipolar with more negatively-charged spores (Buller 1909; Gregory 1957; Saar 2013; Saar and Salm 2014).

Depending on individual spore charges and on relative distances (Fig. 3), electrostatic charged plastic lids of the Petri-dishes may thus differentially attract the also loaded spores. This can then explain why in our experiments mostly only parts of the total spores released from a fruiting body are directed toward the plastic lids positioned above mushrooms (Table 1) and why with higher distances between lids and mushrooms the number of spores attached to the lids decreased in logarithmic fashion (Fig. 3). Overall charges of spores correlate little with spore sizes and actual spore charges are also independent on spore emission rates from mushrooms (Saar and Salm 2014). This is also reflected in our results on different species as shown in Fig. 3. Spore sizes have in contrast been shown to influence sizes of Buller’s drops, the spore velocity upon ejection from the sterigmata, the length of the move vertically through a mushroom airspace before being braked (Stolze-Rybczynski et al. 2009; Fischer et al. 2010a), and the distance of spore deposition from the source (Norros et al. 2014), parameters which appear all be not of primary relevance to the electrostatic attraction and attachment of the spores to plastic lids reported here.

### *Incidence of basidiospore charging in* Agaricomycetes

In this study, basidiospores of in total 66 species with open hymenia and ballistospore propulsion mechanism (from 36 genera, 19 families and 5 orders) did accumulate against gravity in plastic lids and we failed only for four species with each one mushroom tested (*H. virginea*, *H. eburnus*, *L. saeva*, *R. fibula*) to proof such effect. Charging of ballistospores appears thus to be a widely distributed property in the *Agaricomycetes*. This confirms former observations by Saar and Salm (2014). These authors concluded from own and literature data for 43 species, 33 genera, 23–25 families and 9 orders of the *Agaricomycetes* that their ballistospores exhibit electric loads. While we used in our studies in the majority species of the *Agaricales* and to less extend species from the *Boletales* and other orders (Table 2), Saar and colleagues analysed the electric properties of larger species ranges of *Polyporales* and *Boletales* with poroid hymenia, in addition to species from the *Agaricales*, *Russulales* and others (Saar 2013; Saar and Parmasto 2014; Saar and Salm 2014). Considering shared species between the different studies, evidence for ballistospore electric charging is now available for over 100 different species (i.e. 103 or 104 species) of the *Agaricomycetes*.

As we have shown here, charging is not only confined to ballistospores of *Agaricomycetes*. The spores of earthstars which lost the mechanism of ballistospory (Hibbett et al. 1997; Wilson et al. 2011) were also attracted by plastic lids once they were manually pushed into the air. They attached even stronger to the plastic lids than the spores of other *Agaricomycetes*. When trying to brute-force them into solution, they repeatedly formed a dense layer as a cover over the surface of a sphere of 200 µl water. The water ball with the spores on the outside repeatedly burst to slip away from underneath the spore layer which remained then as dry dark brown spot (about 7 mm in *Ø*) of densely packed spores strongly attached to the plastic surface of the lid (our unpublished observations).

### Microbial contaminations in spore solutions

We have demonstrated that basidiospore transfer from upside-down incubated mushrooms with open hymenia to the plastic lids depends on an active ballistospore discharge mechanism (Table 1). This suggested that producing spore prints from up-flying spores would be more selective to the basidiospores than the usual spore prints collected underneath wild mushrooms which tend to be mixed with fallen spores and cells of other microbes. This assumption was tested by plating spore solutions from classical prints and spore solutions collected from plastic lids positioned above upside-down incubated mushrooms (Table 4; Figs. 5, 6). In contrast to conventional spore prints, presence of yeasts or spores from other filamentous fungi which need animal vectors or air flow for their movements was rarely detected in plating tests of spore solutions which were collected from plastic lids positioned above reversed incubated mushrooms (at maximum 10% of samples were contaminated). Bacteria were in somewhat higher frequency present (in up to 22% of total samples of an individual test series), possibly because they may at times be flying directly with a basidiospore. However, there are possible measures against bacteria through addition of suitable antibiotics to the growth media (Fig. 5; Table 4). Further, accumulation of larger numbers of bacterial cells can be precluded by shorter incubation times applied for basidiospore collection (Fig. 6). Already 2–4 h of incubation are sufficient to collect 10^4^–10^5^ spores from mushrooms in the plastic lids (Fig. 2) with no or exceptionally a few bacteria being present in the spore solutions (Fig. 6). Spore ejection rates in our study (Fig. 2; see Table 1 for total ejection figures) correlate with ratios of ballistospore ejection/h from mushrooms reported by Buller (1909, 1922) and to ratios which can be calculated from the spore emission data/s × cm^2^ compiled in Saar and Salm (2014).

Basidiospores secrete hygroscopic hexoses and alcohols (mannitol) localized at their hilar appendices for the assembly of Buller’s drops and at their adaxial shallow dents as hygroscopic compounds for the formation of liquid films, both of which are required for rapid spore catapulting upon their fusion (Goates and Hoffmann 1986; Webster et al. 1989, 1995; Turner and Webster 1995). These organic metabolites together with inorganic ions like phosphate, sodium and potassium are transferred with the liquids spread over the propelled-off spores onto the lids of the Petri-dishes where they might act further hygroscopically to attract more water to the spores (Elbert et al. 2007; Hassett et al. 2015). Eventually, the metabolites might be used for growth of any microbial contaminants happened to be present in the liquid droplets in which basidiospores amplify on the plastic lids during mushroom incubation (Fig. 1). Such microbial growth effect can explain the explosive sudden increase in bacterial cells which was observed more often in spore samples collected after longer incubation times of mushrooms (Fig. 6).

Accordingly, earlier spore harvests can reduce the chance for unwanted bacterial transfer and the time for possible proliferation of contaminants. For later use after a time of storage, drying off the liquid and storing the basidiospores under dry conditions on the sterile plastic lids is also a possible measure of avoidance. Earlier harvest times furthermore reduce the danger of undetected small animals to creep out of mushrooms and to crawl over and contaminate with other microbes the spore collections assembled over the time above the mushrooms on the plastic lids. Such crawling of initially overlooked insect larvae over the spore prints in the plastic lids has indeed been noticed by us on two occasions (not further documented).

### Spore charges in applications

Charging of fungal spores and any potentially connected functions (e.g. in support for spores to serve as nuclei for raindrops; Elbert et al. 2007; Hassett et al. 2015) are understudied and generally little understood (Webster et al. 1988; Wargenau et al. 2011, 2013; Saar 2013; Saar and Parmasto 2014; Saar and Salm 2014). In line, practical use of charging of spores and spore behaviour in electric fields has so far only seldom been made of. However, guarding of bookshelves with an electric field screen has been tested to successfully protect old valuable books in library stack rooms against mold infection by airborne spores (Takikawa et al. 2014) and electrostatic dust collectors were applied in French archives to study their fungal allergenic potentials (Roussel et al. 2012). Spores in buildings and rooms distribute unevenly, among as factors behind by being influenced by electric and magnetic devices (Anaya et al. 2015, 2016). Knowledge on fungal spore distributions in buildings as in outside environments can come through application of electrostatic dust collectors (Normand et al. 2016) and electrostatic precipitators might be used (Han et al. 2011). An electrostatic nursery shelter and an ozone-generative electrostatic spore precipitator were reported to protect tomato plants in hydroponic culture and in open-window greenhouses against fungal pathogens (Shimizu et al. 2007; Kakutani et al. 2012; Takikawa et al. 2016) and an electrostatic spore collector has been applied to collect conidia of the barley pathogen *Blumeria graminis* (Moriura et al. 2006). While our experimental set-up here is simple and easy to apply by using electrostatic features intrinsic to the plastic lids of Petri-dishes to selectively collect basidiospores from mushrooms, our observations offer the possibility to also develop technically more sophisticated devices for basidiospore collection.

## Abbreviations

AB: antibiotics
*Ø*: diameter
RT: room temperature
